# Impact of Teleworking Practices on Presenteeism: Insights from a Cross-Sectional Study of Japanese Teleworkers During COVID-19

**DOI:** 10.3390/bs14111067

**Published:** 2024-11-07

**Authors:** Yuichiro Otsuka, Osamu Itani, Suguru Nakajima, Yuuki Matsumoto, Yoshitaka Kaneita

**Affiliations:** 1 Division of Public Health, Department of Social Medicine, Nihon University School of Medicine, Tokyo 173-8610, Japan; itani@iuhw.ac.jp (O.I.); nakajima.suguru@nihon-u.ac.jp (S.N.); yuuki_m@med.kurume-u.ac.jp (Y.M.); kaneita.yoshitaka@nihon-u.ac.jp (Y.K.); 2 Department of Nursing, School of Medicine, Kurume University School of Nursing, Kurume 830-0003, Japan

**Keywords:** presenteeism, teleworking, Japan, cross-sectional studies, efficiency

## Abstract

Few studies have examined the relationship between teleworking practices and presenteeism. This study determined the association between teleworking practices and presenteeism among teleworkers in Japan. A cross-sectional online survey was administered to 2687 teleworkers from five companies in Japan, collecting data on demographic variables, teleworking practices, frequency and duration of teleworking, presenteeism, and various lifestyle- and health-related factors. A logistic regression analysis was performed. Teleworkers with full-time employment and less teleworking experience exhibited higher presenteeism rates. Key practices negatively associated with presenteeism included creating a dedicated workspace, chatting with colleagues, and setting daily work goals. Gender differences were significant: for men, additional practices, such as determining their work hours, were beneficial; while for women, chatting with colleagues was particularly important. A sensitivity analysis indicated that specific teleworking practices can mitigate presenteeism. Certain teleworking practices, such as creating a workspace, chatting with colleagues, and setting work goals, were associated with lower presenteeism among teleworkers. These findings highlight the need for organizations to support telework ergonomics, promote social interaction, and encourage goal setting to enhance teleworker productivity and health. Training for employees and supervisors to raise awareness of their own and their subordinates’ health while teleworking is advised.

## 1. Introduction

Teleworking involves performing job duties from locations outside the traditional office setting, such as from one’s home or other sites, frequently utilizing information communication technology to complete tasks, and interacting with colleagues within and outside the organization [1]. In 2018, the Japanese government promoted a “work style reform” to improve long working hours and inflexible work styles, promote work-life balance, ensure the labor participation of women and older adults, and increase productivity [2]. Telework—promoted as one such measure [3]—spread rapidly worldwide from the beginning of February 2020 during the coronavirus disease 2019 (COVID-19) pandemic. Japan has a lower rate of telework implementation compared to other developed countries owing to cultural factors [4]. Even in 2023, as the COVID-19 pandemic began to recede, the telework adoption rate among Japanese companies remained at approximately 25–40% [5]. Thus, telework is considered a work style in Japan. In a recent Japanese survey, the introduction of telework was more common in companies that adopted a performance-based system compared to those following the traditional seniority system [4].

Teleworking has multifaceted implications for individuals, organizations, and society. Its advantages include reduced commuting time, reduced informal communication, increased autonomy and flexibility, increased employee independence and commitment to the organization, higher productivity, and better work-life balance [4,6,7,8,9]. However, its disadvantages include work-life boundary issues, social isolation, lack of communication with colleagues, presenteeism, difficulty in evaluating work performance, and poor access to organizational resources [7,9,10,11,12].

Presenteeism is defined as a performance loss that employees experience in the workplace owing to illness or other issues [13]. This phenomenon has a significant detrimental effect on employee productivity, frequently precipitating an increase in sick leave [14]. Several studies have investigated the association between telework and presenteeism [9,11,15,16]; however, their results have been inconsistent. The following mechanisms were considered as negative outcomes. First, the psychosocial safety climate, organizational environment, and organizational practices that prioritize and support employees’ psychological health and safety play crucial roles in reducing presenteeism among teleworkers by lowering psychological demands [17]. A longitudinal study suggested that excessive psychological demands on teleworkers may lead them to view their organization as less supportive of their mental health and overall well-being [17]. Second, teleworking can lead to a blurring of boundaries between work and personal life, potentially resulting in increased presenteeism [18]. Without the physical separation of work and home, employees may struggle to disconnect from their jobs—leading to extended working hours and a constant sense of being present or available for work. Third, the reduction in absenteeism owing to telework may be a health trade-off for employees who continue to telework when they are sick, thereby promoting presenteeism [11]. Workers with poor health conditions may choose to telework when they are sick to avoid absenteeism; however, if they choose to continue working, their job performance may be suboptimal, and their recovery delayed. The positive outcomes included the following mechanisms of increased productivity: teleworkers can work during their most productive hours and are less distracted by colleagues, which also increases their life satisfaction [15].

The factors related to presenteeism among teleworkers are passive employees’ teleworking preference, high intensity and longer working hours, shorter commuting time for on-site work, low ability to detach from work, less supervisor support, and communication difficulties [11,15,19,20,21]. A survey before COVID-19 demonstrated that reduced communication with colleagues, low supervisor support, and the suitability of the working place at home were negatively associated with presenteeism [19]. These factors lead to social isolation, with participants reporting increased stress perception and passive coping strategies [22]. Furthermore, socially isolated individuals exhibited less efficient physiological repair and maintenance, which potentially exacerbated their health issues and increased their likelihood of presenteeism [22].

Gender differences exist in the association between teleworking and presenteeism [23]. Women engaged in home-based telework are more likely than men to concurrently perform multiple roles, such as childcare and housework, and experience conflicts between work and home [24]. Moreover, female remote teleworkers experienced more workload, emotional exhaustion, workaholism, depression, and stress than men, along with higher levels of relaxation and lower levels of loneliness [25].

While much research has examined the effects of teleworking—such as reducing work-family conflict, improving job performance, and addressing professional isolation—less attention has been given to the specific strategies teleworkers use to manage their telework. Research on telework strategies is primarily grounded in boundary theory, which focuses on how individuals establish or dismantle boundaries between their work and personal life [18,26]. Quantitative research on telework strategies is limited, and it has often focused on specific aspects such as boundary management, self-control, or social interaction. A study of 548 German teleworkers found that a conducive work attitude and maintenance of connections were positively associated with work performance, whereas boundary-related telework practices were not associated with work performance rather than presenteeism [18]. This study was limited by its small sample size and was restricted to the cultural background of Northern Europe. No study has investigated these relationships in Japan. Therefore, we conducted a cross-sectional study including a significant number of teleworkers in Japan to determine which teleworking practices are associated with presenteeism in general by gender. Our findings may provide data to improve teleworking presenteeism for occupational health management.

## 2. Materials and Methods

### 2.1. Study Design

An online cross-sectional survey was administered targeting five companies in Japan, including a medical materials supplier, IT firms, and companies from the electronic magazine publishing industry. The target population resided in the Tokyo Metropolitan area, which has a population of approximately 37 million. The sample size was calculated as a minimum of 1537 persons, assuming a 95% confidence level, a 2.5% margin of error, and a population ratio of 0.5. First, participants were recruited from the human resources department of each company via email. The email was not distributed to dispatch employees and contractors, as the companies’ self-survey policies do not cover these categories of workers. Second, the respondents gained survey access via an embedded web link. The link explained the research objectives to the participants before they took the survey. Consent to participate was obtained through a web-based response received in electronic form; those who did not consent could not access or respond to survey items. As this was an anonymous study that used ID numbers as a proxy, it was possible to withdraw consent after responding, resulting in the responses being deleted. This study was conducted in accordance with the Declaration of Helsinki and its current modified version. The survey was administered between December 2021 and January 2022.

The Government of Japan declared a state of emergency four times between April 2020 and September 2021 in many regions of the country. The government asked the public and businesses to reduce the number of people coming to work through teleworking, shorten the opening hours of restaurants, refrain from going out unnecessarily at night, and limit the number of people at events. In November 2021, the government announced that more than 70% of the total population had completed their second dose of the COVID-19 vaccination. Furthermore, it reported that infection levels continued to be the lowest since the summer of 2020 [27]. Therefore, the government’s policies on countermeasures against COVID-19 were revised, and restrictions on eating, drinking, and participating in events, outings, and travel were relaxed under the circumstances [27].

### 2.2. Measures

The online questionnaire contained questions regarding age groups, gender, education history, family members, company position, employment status, work hours (hours/month), holidays (days/month), questions related to telework (teleworking practices, frequency and duration of teleworking, etc.), presenteeism, average sleep duration/weekday, lifestyle habits (smoking and alcohol consumption), non-communicable diseases, and mental health.

#### 2.2.1. Teleworking Practices

The participants were asked how frequently they engaged in the following activities during teleworking: (1) maintaining a constant wakeup time and bedtime, (2) grooming, (3) creating their workspace, (4) receiving sunlight, (5) exercising, (6) determining their work hours, (7) chatting with colleagues, (8) setting daily work goals, (9) avoiding the use of business devices after worktime, (10) listening to music, (11) eating well, (12) talking to family or friends, (13) taking a break, and (14) doing housework. The responses were provided on a 5-point Likert scale ranging from 1 (never) to 5 (always); “often” and “always” were considered affirmative answers. These telework practices were original but based on the extant scientific literature [28,29] and the Ministry of Health, Labour and Welfare’s website.

#### 2.2.2. Frequency and Duration of Teleworking

The frequency of teleworking was assessed using a single item: “How often did you telework (work outside your conventional workplace) in the previous month?” Responses were provided in one of the following categories: “never”, “less than 20% per month”, “20–49% per month”, “50–79% per month”, “80–99% per month”, or “always”.

The duration of teleworking was assessed using a single item: “How long have you performed telework?” Responses were provided in one of the following categories: “less than 3 months”, “3–5 months”, “6–11 months”, “1–3 years”, “3–5 years”, or “over 5 years”. Owing to the small number of responses obtained, the categories “3–5 years” and “over 5 years” were merged into “over 3 years”.

#### 2.2.3. Presenteeism

Presenteeism was the primary outcome measure assessed using the Japanese version of the World Health Organization Health and Work Performance Questionnaire (WHO-HPQ) short version [30,31]. Its reliability and validity were approved by the WHO [32]. It is a single-item questionnaire, with data collected over the past four weeks: “On a scale of 0–10, where 0 is the worst job performance anyone could have at your job and 10 is the performance of a top worker, how would you rate your overall job performance on the days you worked during the past four weeks?” [31]. The score is calculated by multiplying the respondent’s answer by 10, resulting in a range from 0 to 100. Lower scores indicate a lower level of work performance. We defined the cutoff score for presenteeism as 40 based on a previous Japanese study [31]. The area under the curve (AUC) values of the cutoff scores of absolute presenteeism for the prediction of absence due to mental disease was 0.708, with a sensitivity of 50.0% and a specificity of 81.4% [31].

#### 2.2.4. Covariates

Educational history was classified into three categories: (1) high school or vocational school graduate; (2) 4-year college graduate; and (3) obtained a master’s or doctoral degree. The item regarding family members was as follows: “How many people in your household, including yourself, are living with you?” Single, double, and triple combinations were defined. Five categories were defined for the respondent’s position in the company: (1) individual contributor, (2) Section Chief, (3) Division Manager, (4) Department Manager, and (5) Others. Employment status was classified as regular or irregular. Work hours observed for the previous month were classified using the following options: <140 h; ≥140 but <160 h; ≥160 but <180 h; ≥180 but <200 h; ≥200 but <220 h; ≥220 but <240 h; and ≥240 h. Holidays (days/month) included the following options: <4 days, 4–7 days, 8–11 days, and ≥12 days. Smoking status was assessed using the following four options: daily, sometimes, quit smoking, and never. We used “daily” and “sometimes” for current smokers. Alcohol consumption was assessed using the following items: “How many days per week do you consume alcohol on average?” (never, 1 day, 2–4 days, 5–6 days, everyday) and “How many cups of sake do you consume in one session of alcohol consumption?” (less than 1, 1–2 cups, 2–3 cups, >3). One cup (180 mL) of sake was equivalent to 180 mL of 14% alcohol. Daily alcohol consumption was calculated based on the frequency and amount of alcohol consumption, and participants with an alcohol intake of ≥40 g/day (for men) or ≥20 g/day (for women) were defined as having excessive alcohol consumption. The average weekday sleep duration was categorized into the following options: <5 h; ≥5 but <6 h; ≥6 but <7 h; ≥7 but <8 h; and ≥8 h. Those who answered “yes” to the following question were defined as having noncommunicable diseases: “Do you visit a hospital or clinic for treatment of non-communicable diseases?” Mental health was assessed using the 6-item Kessler Scale (K6) [33]. This scale is used to screen for depression, anxiety, and other mental health disorders. The reliability and validity of the scale’s Japanese version have been reported [33]. The scores ranged from 0 to 24, and a score of ≥9 indicated poor mental health [33].

### 2.3. Data Analysis

First, participants’ demographic information was reported and analyzed using a chi-squared test (nominal scale), *t*-test (continuous variables), or one-way analysis of variance (ordinal scale). Second, we compared teleworking practices with presenteeism using a chi-squared test. Third, multivariate logistic regression analyses, with teleworking practices as dependent variables, were conducted to explore participants’ teleworking practices and presenteeism. Model 1 was adjusted for age groups and gender. Model 2 was further adjusted for company, family members, education, working time (hours/month), holidays (days/month), the frequency, and period of telework. Model 3 was further adjusted for employment status, alcohol status, smoking status, sleep duration, mental health, and having disease. In Model 3, stratified analysis was conducted by gender and managerial status. These variables have been reported in previous studies [9,34]. Models 1 to 3 were designed to incrementally isolate the effects of teleworking practices on presenteeism by controlling for demographic, employment, and health-related variables. This tiered approach underscores the robustness of the findings, illustrating that specific telework practices consistently mitigate presenteeism across varied related factors. A sensitivity analysis was performed using a multiple linear regression model with the presenteeism score as the objective variable, without using a cutoff value. Statistical inferences were predicted using a significance threshold of *p* < 0.05; all the tests were two-tailed. We used Stata software (version 18.0; StataCorp, College Station, TX, USA) for all analyses.

### 2.4. Ethical Considerations

This study was approved by the Ethics Committee of Nihon University School of Medicine (Protocol Number: P21-08-0). Informed consent was obtained from all the respondents.

## 3. Results

### 3.1. Participants

Overall, 3015 workers from the five companies responded to the survey. We excluded 231 participants owing to non-teleworking and missing data on telework-related questions (N = 97). Thus, 2687 workers were included in this study.

### 3.2. Descriptive Statistics

This study included 1515 men (56.4%), 1162 women (43.2%), and 10 persons who identified as others (0.4%). Table 1 presents the participants’ descriptive statistics. The 25–29 years age group accounted for the highest proportion of participants. Most participants had a regular employment status, college education history, and received 8–11 holidays per month. Participants with presenteeism exhibited significantly higher percentages of regular employment and poor mental health; they were from younger age groups, held more junior positions, had longer work duration, moderate alcohol drinking, and shorter sleep duration.

### 3.3. Association Between the Frequency and Experience of Telework and Presenteeism

Table 2 presents the association between the frequency and experience of teleworking and presenteeism, which was frequently observed among full-time teleworking workers with limited experience.

### 3.4. Association Between Teleworking Practices and Presenteeism

Table 3 elucidates the percentages of teleworking practices according to presenteeism status. Participants with presenteeism tended to adopt fewer teleworking practices than those without it. Significant associations were observed for most teleworking practices, except for receiving sunlight, avoiding the use of business devices after work time, listening to music, taking a break, and doing housework. Table 4 presents the results of the logistic regression analysis of the association between teleworking practices and presenteeism. All the models exhibit similar results. In Model 3, creating their workspace (odds ratio [OR] = 0.67 [95% confidence interval (CI), 0.50 to 0.90], *p* = 0.008), chatting with colleagues (OR = 0.42 [95% CI, 0.26 to 0.70], *p* = 0.001), and setting daily work goals (OR = 0.61 [95% CI, 0.45 to 0.84], *p* = 0.002) were negatively associated with presenteeism.

### 3.5. Gender Differences in the Association Between Teleworking Practices and Presenteeism

**Figure 1:**
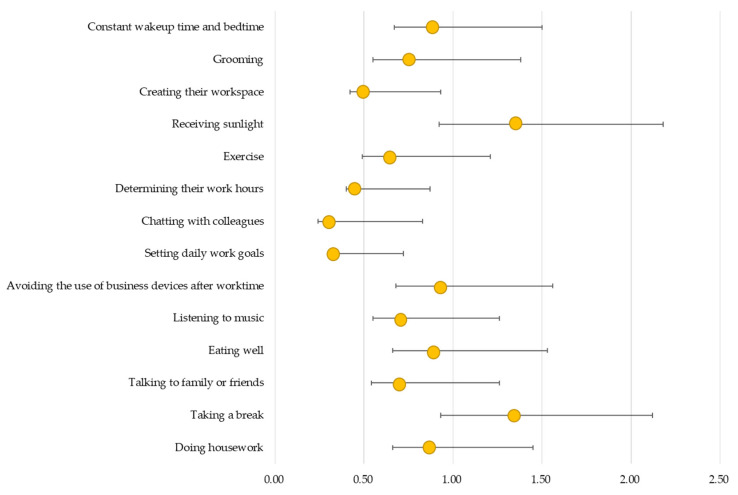
Association between teleworking practices and presenteeism among male teleworkers in Japan (N = 1515). Adjusted for age group, company, employment status, family member, education, working time (hours/month), holidays (days/month), alcohol status, smoking status, sleep duration, mental health, having disease, and the frequency and duration of telework.

**Figure 2:**
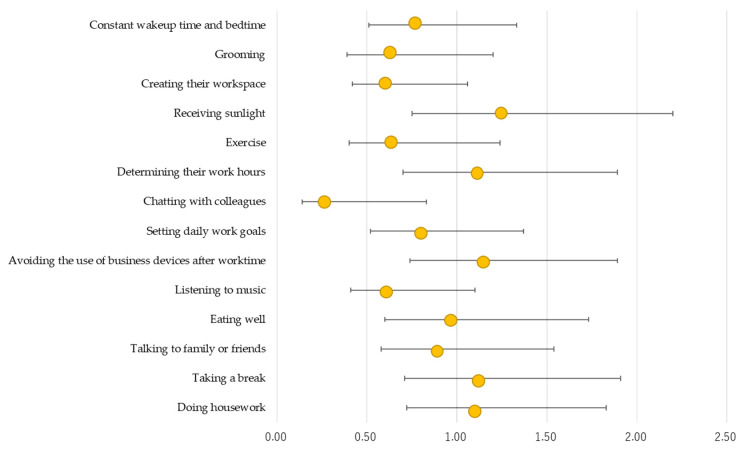
Association between teleworking practices and presenteeism among female teleworkers in Japan (N = 1162). Adjusted for age group, company, employment status, family member, education, working time (hours/month), holidays (days/month), alcohol status, smoking status, sleep duration, mental health, having disease, and the frequency and duration of telework.

Figure 1 and Figure 2 present the results of the logistic regression analysis of the associations between teleworking practices and presenteeism by gender. For men, creating their workspace (OR = 0.62 [95% CI, 0.42 to 0.93], *p* = 0.021), determining work hours (OR = 0.59 [95% CI, 0.40 to 0.87], *p* = 0.008), chatting with colleagues (OR = 0.45 [95% CI, 0.24 to 0.83], *p* = 0.010), and setting daily work goals (OR = 0.48 [95% CI, 0.31 to 0.72], *p* = 0.001) were negatively associated with presenteeism (Figure 1). However, for women, only chatting with colleagues (OR = 0.35 [95% CI, 0.14 to 0.83], *p* = 0.017) was negatively associated with presenteeism (Figure 2).

### 3.6. Position Differences in the Association Between Teleworking Practices and Presenteeism

Supplementary Figures S1 and S2 present the results of the linear regression analysis of the associations between teleworking practices and presenteeism by position. For non-managed positions (individual contributor and section chief), creating their workspace (OR = 0.60 [95% CI, 0.42 to 0.84], *p* = 0.021), chatting with colleagues (OR = 0.49 [95% CI, 0.29 to 0.82], *p* = 0.007), and setting daily work goals (OR = 0.60 [95% CI, 0.42 to 0.84], *p* = 0.003) were negatively associated with presenteeism (Supplemental Figure S1). For managed positions (division manager and department manager), chatting with colleagues (OR = 0.19 [95% CI, 0.04 to 0.86], *p* = 0.031) and listening to music (OR = 0.33 [95% CI, 0.12 to 0.93], *p* = 0.036) were negatively associated with presenteeism (Supplemental Figure S2).

### 3.7. Sensitivity Analysis of the Association Between Teleworking Practices and Presenteeism Scores

Among the participants, consistent wakeup time and bedtime (β = 1.91 [95% CI, 0.28 to 3.54], *p* = 0.022), creating their workspace (β = 2.68 [95% CI, 0.96 to 4.40], *p* = 0.002), chatting with colleagues (β = 5.29 [95% CI, 3.37 to 7.21], *p* < 0.001), setting daily work goals (β = 5.43 [95% CI, 3.90 to 6.96], *p* < 0.001), and listening to music (β = 2.12 [95% CI, 0.51 to 3.73], *p* = 0.010) were associated with the presenteeism score. Similar trends were observed for gender. However, avoiding the use of business devices after worktime (β= −0.20 [95% CI, −0.34 to −0.07], *p* = 0.004) was negatively associated with work engagement. Creating their workspace was significant for men and *listening to music* was significant only for women (Supplementary Table S1).

## 4. Discussion

To the best of our knowledge, this is the first study to examine the association between teleworking practices and presenteeism in Japan. The main findings are as follows: (1) The implementation of fully remote, high intensity teleworking experiences was associated with presenteeism. (2) Teleworking practices, such as creating a workspace, chatting with colleagues, and setting daily work goals, were negatively associated with presenteeism. (3) Gender differences were observed in practices related to presenteeism.

### 4.1. Association Between Teleworking Frequency and Experience and Presenteeism

Teleworking frequency and experience were associated with presenteeism. Based on previous research, this supports our assumption that full-time teleworking negatively affects presenteeism [35]. In fully remote conditions, workers may be able to continue working even if they become ill, which may increase their presenteeism. The lack of face-to-face interaction and social isolation with increasing telework intensity may contribute to presenteeism. A web-based cross-sectional survey of 542 professionals with previous or current experience in home-based teleworking reported that teleworking experience was positively associated with self-reported productivity [36]. These findings suggest that the more time workers spend teleworking, the more likely they are to adapt to the environment and avoid presenteeism.

### 4.2. Implications of Key Findings

Teleworkers with presenteeism tend not to perform many teleworking activities compared to those without presenteeism. However, only three teleworking practices were associated with presenteeism in our multivariate analysis, indicating that creating an individual workspace is negatively associated with presenteeism. Supporting this finding, a previous review found that ergonomically designed workspaces positively affect teleworkers’ health [8]. Teleworkers frequently establish their own workspaces and engage in unsafe behaviors such as working on couches or other uncomfortable workspaces [37]. Several studies have reported that workers who were forced to adopt teleworking during the COVID-19 pandemic did not have a dedicated place to work at home or sufficient furniture and workspace [38]. Additionally, numerous teleworkers do not have sufficient awareness or knowledge of ergonomic and safety issues in their homes [39]. Moreover, several companies lack adequate regulations or policies regarding remote workspace settings or ergonomic assessments [40]. For example, a cross-sectional study of 934 workers during the COVID-19 pandemic demonstrated that the lack of suitable places for teleworking was associated with ergonomic and psychosocial risks and musculoskeletal problems [40]. These problems can result in presenteeism [41]. Therefore, creating comfortable workspaces may precipitate teleworker presenteeism, and organizations must implement teleworking ergonomic training to improve workers’ knowledge of and attitudes toward ergonomic resources.

A systematic review has suggested that social isolation is associated with presenteeism [16]. Teleworkers are expected to feel socially isolated because they spend long hours alone [42]. This study revealed that chatting with colleagues was negatively associated with presenteeism. Similarly, a cross-sectional study of Japanese civil servants before the COVID-19 pandemic reported that a higher perceived level of social support from supervisors and colleagues exhibited a protective effect against presenteeism [43]. Thus, these results suggest that chatting with colleagues may be a strategy for preventing social isolation. Moreover, our study revealed that talking to family members rather than friends was not associated with presenteeism. However, a cross-sectional study of workers in Japan reported that the change in time spent talking with family, not friends, was positively associated with absolute presenteeism scores during the COVID-19 pandemic [44]. The major difference between this previous study and the current study is the presence of mental health adjustments and the possibility that conversations with family and friends may prevent presenteeism through mental health.

Consistent with previous studies [18], setting daily work goals was negatively associated with presenteeism. Based on goal-setting theory, specific and challenging goal setting results in higher performance by encouraging individuals to do their best [45]. As specific goals vary in difficulty, goal specificity alone does not necessarily precipitate high performance. However, as long as performance is completely controllable, goal specificity reduces performance variability by mitigating the ambiguity regarding what needs to be accomplished.

### 4.3. Other Teleworking Practices

Turning work business devices on and off, exercising, eating well, and maintaining a constant life rhythm were not significantly associated with presenteeism in the current multivariate analysis. These practices are considered boundary management strategies [28]. This is consistent with previous studies [18,28], as the global implementation of boundary management strategies is not associated with work productivity. However, these practices are recommended in the Guidelines for the Promotion of Appropriate Introduction and Implementation of Telework in Japan [3]. Although no evidence explains this discrepancy, these practices may undermine teleworker independence, become confounding factors for other practices, or decrease detection power owing to a binary outcome.

Additionally, some studies have identified the association between employee health behaviors such as physical activity, nutrition, and telework. Moreover, teleworkers had a significantly lower risk of malnutrition and inactivity than non-teleworkers [9]. Physical activity and favorable dietary habits are considered a potential practice for avoiding presenteeism [46].

### 4.4. Gender Differences

Gender roles play a significant part in teleworking practices, with home-based telework potentially reinforcing traditional gender roles while simultaneously offering flexibility [47]. Recent research on mandatory teleworking during COVID-19 in Japan found that it did not significantly affect gender role attitudes among dual-career couples but was associated with lower work-family conflict, particularly for women [48]. Chatting with colleagues was a common practice against presenteeism for both genders in the current study. However, other practices were not associated with presenteeism for women. As women’s roles in the household are generally greater than men’s [24], women are reportedly drawn to telework as a means of balancing work and family responsibilities [49]. Thus, women might perform other practices such as determining their work hours daily whether they are aware of it or not. In addition, women were more likely to emotionally communicate with coworkers using text messaging, social media, and video calls, while men restrict their communication to work [50,51]. These findings suggest that while cultural barriers persist, evolving societal needs and external factors are gradually reshaping attitudes towards telework in Japan, with implications for gender dynamics in work and family life.

Our gender-inclusive approach reflects the evolving understanding of gender diversity in the workplace. While the sample size for individuals identifying as non-binary or “other” was small, their inclusion is a step towards understanding telework challenges across all gender identities. Future studies with larger samples should explore how gender diversity affects teleworking practices and presenteeism; this will help to potentially gather unique insights into work-life dynamics and mental health in telework environments.

### 4.5. Other Differences

This study demonstrated that different positions had different practice behaviors during telework with regards to presenteeism. Managers practiced discretion and had an environment where they could listen to music. Non-managers, however, were given tasks and were more likely to set work goals based on those tasks. For example, presenteeism among Chinese workers in Japan was related to health-promoting lifestyles, which vary by job position and other sociodemographic factors [52]. Cultural differences play a role in presenteeism. For example, the prevalence of presenteeism varied significantly between countries, ranging from 3.9 days in Ireland to 22.1 days in Spain over a 12-month period [53]. Chinese employees exhibited higher presenteeism rates and strain levels compared to their British counterparts [54]. Leadership behaviors impacted presenteeism, though their effects varied across countries [53]. However, job stress and supervisory support were consistently correlated with increased presenteeism across cultures [53,54].

### 4.6. Strengths and Limitations

This study has several limitations. First, participant sampling and response collection may have introduced selection bias. The participants were employees of only five Japanese companies in the Tokyo metropolitan area. In addition, the results may be influenced by potential cultural biases unique to Japan and companies functioning within it [4]. Therefore, generalizing these findings to employees from other companies or areas may not be possible. Second, the survey data were obtained using self-administered questionnaires, which may have resulted in reporting bias. However, to reduce recall bias, the recall periods were limited to four and 30 days. Third, compared to the general working population, although the man/woman ratio is not significantly different, the study’s results are difficult to generalize because of the large percentage of the younger generation in this study. Fourth, the study design was cross-sectional; thus, a causal association between teleworking practices and insomnia could not be confirmed. Fifth, this study was conducted during the COVID-19 pandemic, which may have influenced the results. Further studies should be conducted after the pandemic. Despite these limitations, our study’s strengths include the large sample size, inclusion of varied occupations, and use of well-validated scales for presenteeism.

### 4.7. Future Research Directions

From a public health perspective, this study supports the important role of teleworking practices in occupational health management. The teleworking practices discussed in this study are relatively easy to implement in daily work. Future research should adopt longitudinal, qualitative designs to explore these practices deeply, investigate this association in other populations and companies, and examine the mechanisms underlying the observed associations. In addition, future research should examine the relationship between explanatory variables and presenteeism at multiple levels using cross-level analysis.

## 5. Conclusions

Teleworking practices play an important role in presenteeism. Our findings can be useful for devising measures to improve work performance and for informing organizations, supervisors, and occupational hygiene staff regarding the incorporation of adaptive practices among teleworkers. This study highlighted the impact of telework practices on individual productivity and presenteeism, with implications for organizational policy. Given Japan’s shift towards a performance-based evaluation system [4], organizations may benefit from considering how this evolving structure influences telework dynamics and employee health outcomes. The performance-based system may increase pressure on employees which ca exacerbate their presenteeism, thus suggesting the need for balanced evaluation criteria that recognize both productivity and well-being in telework contexts. Organizations should subsidize employees purchasing necessary telework equipment; supervisors and colleagues must prioritize communication more than they would in an onsite setting, and employees themselves need to establish daily work goals. Training for employees and leaders to raise awareness of their own and their subordinates’ health while teleworking and clear guidelines within the organization to deal with presenteeism may also be helpful. Encouraging employees who have been teleworking for a longer period to meet regularly for chats or to join the team on-site is essential. However, more generalized studies assessing the causal association between teleworking practices and presenteeism are required.

## Figures and Tables

**Table 1:** Characteristics of the study participants.

	Non Presenteeism		Presenteeism		Chi-Squared Value	*p*-Value ^1^
	N = 2406		N = 281			
	N	%	N	%		
Gender					2.2	0.332
Male	1348	56.0	167	59.4		
Female	1048	43.6	114	40.6		
Others	10	0.4	0	0.0		
Age group					17.7	<0.001
20–24	148	6.2	25	8.9		
25–29	732	30.4	109	38.8		
30–34	523	21.7	55	19.6		
35–39	395	16.4	38	13.5		
40–44	284	11.8	31	11.0		
45–49	157	6.5	13	4.6		
50–59	134	5.6	10	3.6		
60+	33	1.4	0	0.0		
Regular employment	2345	97.5	281	100.0	7.3	0.007
Education					3.5	0.176
High school or vocational school	265	11.0	21	7.5		
College	1674	69.6	206	73.3		
Graduate school	467	19.4	54	19.2		
Family member					1.5	0.484
Single	838	34.8	92	32.7		
Two	672	27.9	88	31.3		
Over three	896	37.2	101	35.9		
Position					10.6	0.031
Individual contributor	1093	45.4	155	55.2		
Section Chief	834	34.7	78	27.8		
Division Manager	400	16.6	42	14.9		
Department Manager	71	3.0	6	2.1		
Others	8	0.3	0	0.0		
Working duration/month					11.5	0.074
<140	219	9.1	28	10.0		
140–160	323	13.4	52	18.5		
160–180	927	38.5	110	39.1		
180–200	540	22.4	51	18.1		
200–220	272	11.3	22	7.8		
220–240	79	3.3	7	2.5		
>240	32	1.3	6	2.1		
Holidays/month					12.4	0.006
<4 d	175	7.3	15	5.3		
4–7 d	156	6.5	19	6.8		
8–11 d	1792	74.5	192	68.3		
≥12 d	266	11.1	50	17.8		
Poor mental health	418	17.4	118	42.0	97.3	<0.001
Current smoking	311	12.9	34	12.1	0.2	0.693
Moderate alcohol drinking	807	33.5	70	24.9	8.5	0.004
Average sleep duration/weekday					5.5	0.237
<5 h	155	6.4	27	9.6		
5–6 h	606	25.2	70	24.9		
6–7 h	824	34.2	100	35.6		
7–8 h	651	27.1	68	24.2		
>8 h	107	4.4	9	3.2		

^1^ *p*-value was calculated by chi-squared test.

**Table 2:** Associations between frequency and experience of telework and absolute presenteeism.

Telework	Non Presenteeism		Presenteeism		Chi-Squared Value	*p*-Value
	N = 2406		N = 281			
	N	%	N	%		
Frequency					14.0	0.007
1–19%	150	6.2	19	6.8		
20–49%	197	8.2	23	8.2		
50–79%	177	7.4	23	8.2		
80–99%	466	19.4	29	10.3		
100%	1416	58.9	187	66.5		
Experience					11.9	0.018
<3 months	100	4.2	14	5.0		
3–6 months	178	7.4	23	8.2		
6–12 months	271	11.3	50	17.8		
1–3 years	1800	74.8	189	67.3		
Over 3 years	57	2.4	5	1.8		

**Table 3:** Associations between teleworking practices and absolute presenteeism.

Teleworking Practices	Non Presenteeism		Presenteeism		Chi-Squared Value	*p*-Value
	N = 2406		N = 281			
	N	%	N	%		
Constant wakeup time and bedtime	1443	60.0	132	47.0	17.5	<0.001
Grooming	742	30.8	58	20.6	12.5	<0.001
Creating their workspace	1777	73.9	159	56.6	37.3	<0.001
Receiving sunlight	979	40.7	99	35.2	3.1	0.077
Exercise	879	36.5	73	26.0	12.3	<0.001
Determining their work hours	1472	61.2	142	50.5	11.9	0.001
Chatting with colleagues	482	20.0	20	7.1	27.6	<0.001
Setting daily work goals	1092	45.4	75	26.7	35.8	<0.001
Avoiding the use of business devices after worktime	932	38.7	101	35.9	0.8	0.362
Listening to music	799	33.2	80	28.5	2.6	0.109
Eating well	1782	74.1	189	67.3	6.0	0.015
Talking to family or friends	1209	50.2	113	40.2	10.1	0.001
Taking a break	1009	41.9	110	39.1	0.8	0.369
Doing housework	1161	48.3	121	43.1	2.7	0.099

**Table 4:** Association between teleworking behavior and presenteeism among teleworkers in Japan.

Teleworking Practices	Model 1 ^1^			Model 2 ^2^			Model 3 ^3^		
	OR	95% CI	*p*-Value	OR	95% CI	*p*-Value	OR	95% CI	*p*-Value
Constant wakeup time and bedtime	0.88	0.67–1.17	0.378	0.88	0.66–1.17	0.388	0.92	0.68–1.24	0.574
Grooming	0.92	0.66–1.28	0.628	0.86	0.61–1.21	0.387	0.83	0.58–1.17	0.288
Creating their workspace	0.60	0.46–0.79	<0.001	0.63	0.47–0.83	0.001	0.67	0.50–0.90	0.008
Receiving sunlight	1.30	0.95–1.77	0.101	1.31	0.96–1.80	0.092	1.37	0.98–1.91	0.065
Exercise	0.71	0.51–0.98	0.040	0.72	0.51–1.00	0.051	0.85	0.59–1.22	0.375
Determining their work hours	0.83	0.63–1.10	0.196	0.76	0.57–1.01	0.057	0.75	0.55–1.01	0.056
Chatting with colleagues	0.37	0.23–0.59	<0.001	0.39	0.24–0.63	<0.001	0.42	0.26–0.70	0.001
Setting daily work goals	0.57	0.43–0.77	<0.001	0.56	0.41–0.75	<0.001	0.61	0.45–0.84	0.002
Avoiding the use of business devices after worktime	1.11	0.84–1.47	0.470	1.04	0.78–1.38	0.811	1.09	0.80–1.47	0.584
Listening to music	0.81	0.60–1.09	0.157	0.82	0.60–1.10	0.184	0.78	0.57–1.06	0.107
Eating well	0.94	0.69–1.27	0.691	0.94	0.69–1.28	0.690	1.03	0.75–1.43	0.847
Talking to family or friends	0.84	0.63–1.12	0.226	0.75	0.55–1.02	0.069	0.87	0.63–1.20	0.407
Taking a break	1.25	0.93–1.67	0.135	1.23	0.91–1.65	0.174	1.30	0.95–1.77	0.098
Doing housework	1.02	0.78–1.35	0.863	1.04	0.78–1.39	0.773	1.07	0.80–1.44	0.635

^1^ Model 1: Adjusted for age group and gender. ^2^ Model 2: Adjusted for age group, gender, company, family member, education, working time (hours/month), holidays (days/month), and the frequency and duration of telework. ^3^ Model 3: Adjusted for age group, gender, company, employment status, family member, education, working time (hours/month), holidays (days/month), alcohol status, smoking status, sleep duration, mental health, having disease, and the frequency and duration of telework.

## Data Availability

The datasets presented in this article are not readily available because of privacy restrictions.

## Supplementary Materials

The following supporting information can be downloaded at the following: https://www.mdpi.com/article/10.3390/bs14111067/s1. Table S1: Linear regression of association between teleworking behavior and presenteeism among teleworkers in Japan; Figure S1: Association between teleworking practices and presenteeism among without managed teleworkers in Japan; Figure S2: Association between teleworking practices and presenteeism among managed teleworkers in Japan.
