# Supplementary material for: Impact of Teleworking Practices on Presenteeism: Insights from a Cross-Sectional Study of Japanese Teleworkers During COVID-19
Source: Behav Sci (Basel). 2024 Nov 7;14(11):1067. doi: 10.3390/bs14111067 (PMC11591190; doi:10.3390/bs14111067)
Supplement: Supplementary file 1 [file behavsci-14-01067-s001.zip › behavsci-3268461-supplementary/Table S1.pdf]

Supplemental Table S1. Linear regression of association between teleworking behavior and presenteeism among teleworkers in Japan

**Teleworking practices**	**beta**	**95%CI**			**p-value**
Constant wakeup time and bedtime	1.91	0.28	-	3.54	0.022
Grooming	0.09	-1.62	-	1.79	0.918
Creating their workspace	2.68	0.96	-	4.40	0.002
Receiving sunlight	-0.40	-2.15	-	1.35	0.651
Exercise	1.08	-0.69	-	2.85	0.232
Determining their work hours	1.06	-0.57	-	2.69	0.204
Chatting with colleagues	5.29	3.37	-	7.21	< 0.001
Setting daily work goals	5.43	3.90	-	6.96	< 0.001
Avoiding the use of business devices after worktime	-0.65	-2.21	-	0.91	0.415
Listening to music	2.12	0.51	-	3.73	0.010
Eating well	-0.01	-1.80	-	1.77	0.987
Talking to family or friends	1.30	-0.40	-	3.00	0.135
Taking a break	-0.51	-2.14	-	1.12	0.541
Doing housework	-0.53	-2.10	-	1.05	0.510

Adjusted for age groups, gender, company, employment status, family member, education, working time (hours/month), holidays (days/month), alcohol status, smoking status, sleep duration, mental health, having disease, and the frequency and period telework
