# Supplementary figures and images for: Impact of Teleworking Practices on Presenteeism: Insights from a Cross-Sectional Study of Japanese Teleworkers During COVID-19
Source: Behav Sci (Basel). 2024 Nov 7;14(11):1067. doi: 10.3390/bs14111067 (PMC11591190; doi:10.3390/bs14111067)

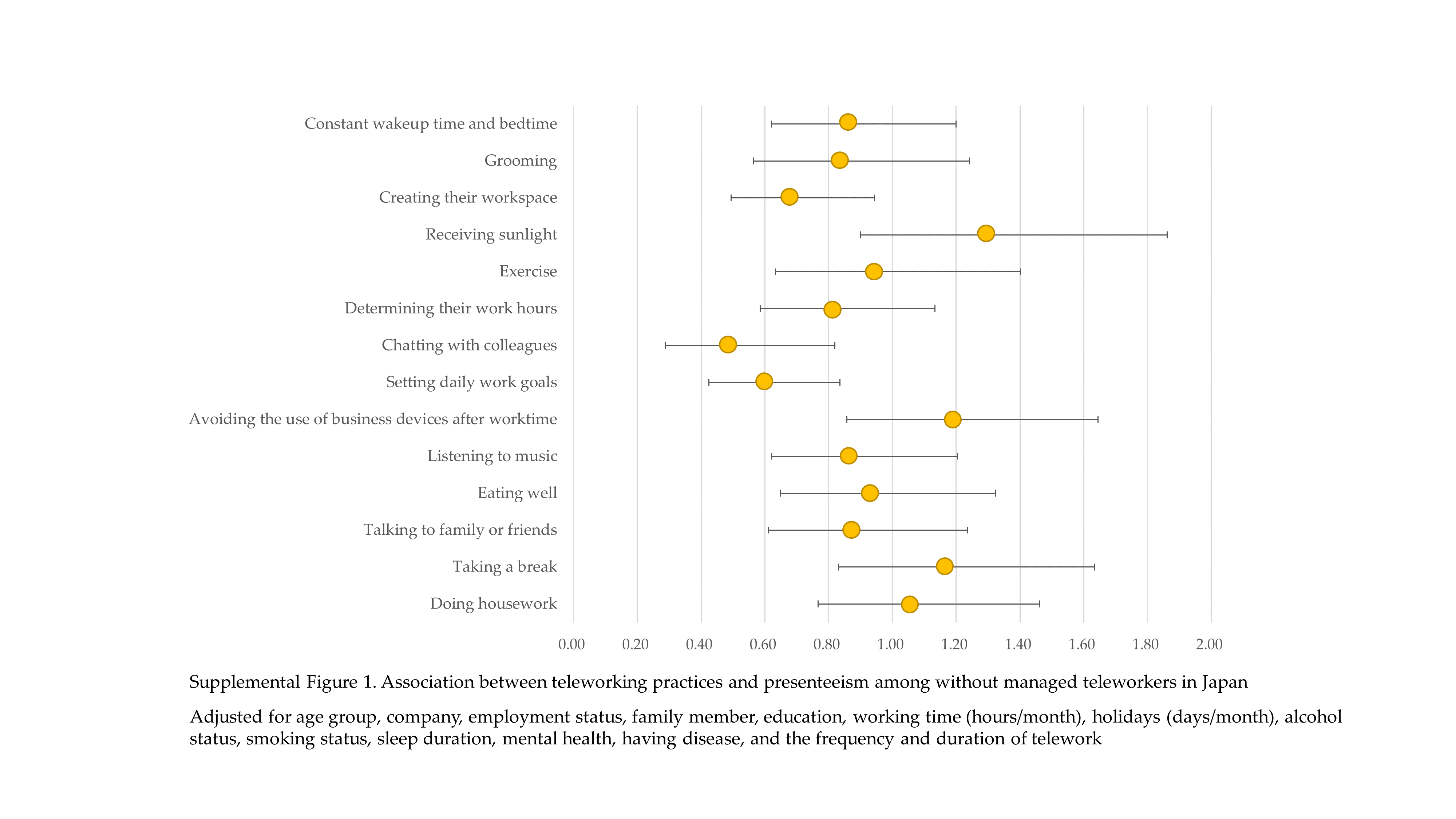

Supplement: Supplementary file 1 [file behavsci-14-01067-s001.zip › behavsci-3268461-supplementary/Figure S1.PNG]

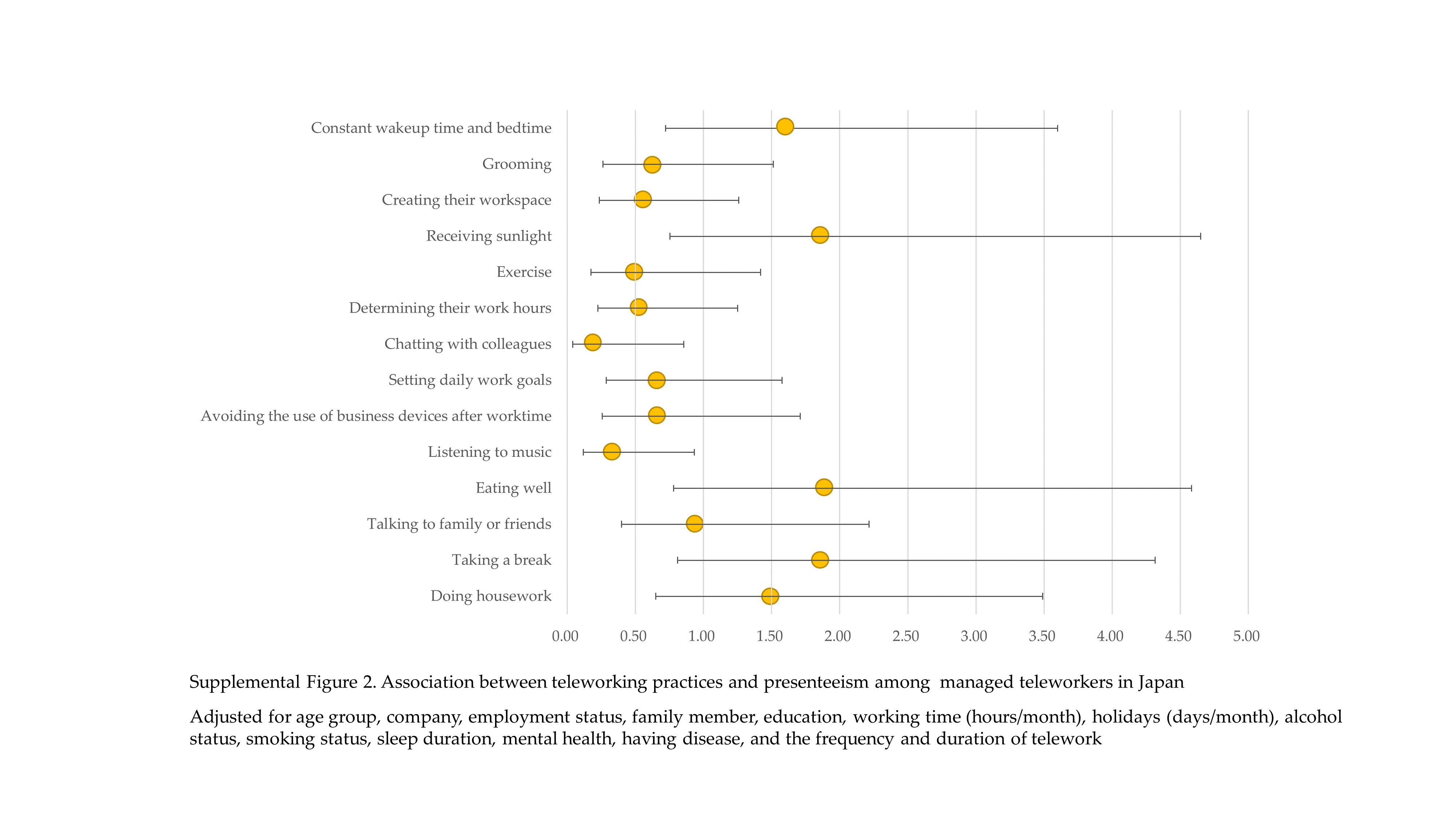

Supplement: Supplementary file 1 [file behavsci-14-01067-s001.zip › behavsci-3268461-supplementary/Figure S2.PNG]
